# Oleacein and Oleocanthal: Key Metabolites in the Stability of Extra Virgin Olive Oil

**DOI:** 10.3390/antiox12091776

**Published:** 2023-09-18

**Authors:** Alexandra Olmo-Cunillera, Maria Pérez, Anallely López-Yerena, Mohamed M. Abuhabib, Antònia Ninot, Agustí Romero-Aroca, Anna Vallverdú-Queralt, Rosa Maria Lamuela-Raventós

**Affiliations:** 1 Polyphenol Research Group, Department of Nutrition, Food Science and Gastronomy, Catalonia Food Innovation Network (XIA), Faculty of Pharmacy and Food Sciences, Institute of Nutrition and Food Safety (INSA-UB), University of Barcelona, 08028 Barcelona, Spain; alexandra.olmo@ub.edu (A.O.-C.); mariaperez@ub.edu (M.P.); nayelopezye@ub.edu (A.L.-Y.); mabuhaab8@alumnes.ub.edu (M.M.A.); avallverdu@ub.edu (A.V.-Q.); 2 CIBER Physiopathology of Obesity and Nutrition (CIBEROBN), Instituto de Salud Carlos III, 28029 Madrid, Spain; 3 Institute of Agrifood Research and Technology (IRTA), Fruit Science Program, Olive Growing and Oil Technology Research Team, 43120 Constantí, Spain; antonia.ninot@irta.cat (A.N.); agusti.romero@irta.cat (A.R.-A.)

**Keywords:** *Olea Europaea*, oxidation, Rancimat, polyphenols, chlorophylls, high-quality, MUFA/PUFA, multivariate analysis

## Abstract

The oxidative stability of extra virgin olive oil (EVOO) depends on its composition, primarily, phenolic compounds and tocopherols, which are strong antioxidants, but also carotenoids, squalene, and fatty acids contribute. The aim of this study was to evaluate the effect of malaxation conditions and olive storage on the composition of ‘Corbella’ EVOO produced in an industrial mill to determine which parameters and compounds could give more stable oils. Although a longer malaxation time at a higher temperature and olive storage had a negative effect on the content of *α*-tocopherol, squalene, flavonoids, lignans, phenolic acids, and phenolic alcohols, the antioxidant capacity and oxidative stability of the oil were improved because of an increase in the concentration of oleacein (56–71%) and oleocanthal (42–67%). Therefore, these two secoiridoids could be crucial for better stability and a longer shelf life of EVOOs, and their enhancement should be promoted. A synergistic effect between secoiridoids and carotenoids could also contribute to EVOO stability. Additionally, ‘Corbella’ cultivar seems to be a promising candidate for the production of EVOOs with a high oleic/linoleic ratio. These findings signify a notable advancement and hold substantial utility and significance in addressing and enhancing EVOO stability.

## 1. Introduction

A serious problem affecting edible oils is lipid oxidation, a major cause of deterioration of chemical, sensory, and nutritional properties. Extra virgin olive oil (EVOO) is highly resistant to oxidative degradation, due to a low content of polyunsaturated fatty acids (PUFAs) and high levels of monounsaturated fatty acids (MUFAs), as well as the presence of phenolic compounds and tocopherols [1]. Nevertheless, the variable composition of EVOOs means their resistance to oxidative deterioration also differs.

The main factors affecting the fatty acid (FA) profile and triacylglycerol composition of EVOO are the climate in which the olives are cultivated, their cultivar, and stage of maturity when harvested [1]. Parameters of interest are the ratios of MUFA/PUFA and oleic/linoleic acids, which give information about the oxidative stability and rancidity of the oils [2]: the higher the values, the more stable and less rancid they are. The two ratios are correlated, as oleic acid is the main MUFA and linoleic acid the principal PUFA in olive oil. As the autoxidative stability of oleic acid is 10-fold higher than that of linoleic acid [3], olive oils with high oleic and low linoleic acid content are better from both a nutritional and technological standpoint. Accordingly, the generation of new olive cultivars producing oils with a high oleic/linoleic ratio is a priority in olive breeding programs [2].

The minor unsaponifiable fraction of EVOO contains two main groups of compounds that act as primary inhibitors of oxidation: phenolic compounds and tocopherols. Phenolic compounds are hydrophilic antioxidants only found in olive oils if they are virgin, as they are lost during the refining process. The highest contributors to oxidative stability in EVOO are *o*-diphenols such as hydroxytyrosol and its oleoside forms (oleuropein, oleuropein aglycone, and oleacein) [1,4]. Tocopherols are lipophilic antioxidants that reduce lipid oxidation as well as photooxidation [1]. The major tocopherol in olive oil is *α*-tocopherol, with *β*- and *γ*-tocopherol found in minor amounts. The major constituent of the unsaponifiable fraction in olive oil is squalene, which has a lower antioxidant activity compared to phenolic compounds and α-tocopherol. It acts at low or moderate temperatures, and in combination with *α*-tocopherol and phenolic compounds [1].

Chlorophylls and carotenoids are the pigments responsible for the color of olive oil. In the presence of light, chlorophylls and their derivatives are the most active promoters of photosensitized oxidation in EVOO, contributing greatly to its susceptibility to oxidation [5]. Nevertheless, they show antioxidant effects in the dark [6]. In contrast, carotenoids, especially *β*-carotene, are strong protectors against photosensitized oxidation, acting as singlet oxygen quenchers [5].

Among the principal factors affecting EVOO composition are the cultivar, ripeness, and health of the olive fruits, agroclimatic conditions, the production process, including crushing, malaxation, extraction and filtering, and storage [1]. Maximizing the concentrations of antioxidant components will ensure an oil with higher stability. As the ripening index (RI) of the olives increases, their phenolic content decreases, resulting in oils with lower oxidative stability [4,7,8,9]; likewise, chlorophylls and carotenoids decrease drastically, while the PUFA levels increase [4,8,9]. Furthermore, the storage of olives before oil production increases hydrolytic and oxidative degradation, leading to a depletion in the content of phenolic compounds, tocopherols, and carotenoids, therefore impairing the oil stability, especially when storage is prolonged [10].

In a previous pilot study using an ABENCOR system (Abengoa S.A., Seville, Spain), the effect of the RI and malaxation conditions on the phenolic content of ‘Corbella’ EVOOs was evaluated [7]. Additionally, a targeted metabolic profiling of this ancient olive cultivar was conducted to determine the composition of olives at an early maturation stage [11]. As a continuation of this research, with the aim of understanding and improving oil stability and shelf life, the present study analyzed ‘Corbella’ EVOOs produced in an industrial mill under different malaxation conditions using olives of a similar RI (1 to 1.5). The effect of storing the olives overnight for 17 h at ambient temperature on the EVOO composition and oxidative stability was also evaluated. This is the first time that ‘Corbella’ EVOOs produced in an industrial mill are analyzed to determine the effect of olive storage and malaxation conditions. The study of olive oils produced in industrial mills is always more accurate than studying oils obtained at laboratory scale. Furthermore, as ‘Corbella’ is an ancient cultivar recently reintroduced, more information is needed to understand its oxidative stability, information that can also be useful in understanding other cultivars.

## 2. Materials and Methods

### 2.1. Reagents

*n*-Hexane, 0.5 N sodium methoxide, 14% boron trifluoride–methanol, Trolox, diphenyl-1-picryl-hydrazyl (DPPH), and Folin–Ciocalteu’s reagent were purchased from Sigma-Aldrich (St. Louis, MO, USA); acetic acid, formic acid, methanol, acetonitrile (ACN), *N*,*N*-dimethylformamide (DMF), and tertbutylmethylether (TBME) from Sigma-Aldrich (Madrid, Spain); and sodium chloride (NaCl) and sodium carbonate (Na_2_CO_3_) from Panreac Química SLU (Castellar del Vallès, Spain). Ultrapure water was obtained using a Milli-Q purification system (Millipore, Bedford, MA, USA).

Regarding the standards (≥90% purity), oleocanthal was purchased from Merck (Darmstadt, Germany), and oleacein, oleuropein aglycone, and elenolic acid from Toronto Research Chemical Inc. (North York, ON, Canada). Oleuropein, ligstroside, pinoresinol, gallic acid, vanillic acid, caffeic acid, verbascoside, rutin, chlorophyll a, lutein, *β*-carotene, squalene, and (α)-tocopherol were acquired from Sigma-Aldrich. Apigenin, ferulic acid and *p*-coumaric were obtained from Fluka, and hydroxytyrosol from Extrasynthese (Genay, France). Methyl tridecanoate (C13:0) was used as a standard for the analysis of FAs and was acquired from Sigma-Aldrich.

### 2.2. Samples

The ‘Corbella’ olive samples were all collected on 13 October 2021. The olive orchard is in Valls de Torroella (Barcelona, Catalonia, Spain) which is sited at latitude 41°52′12.9″ N and longitude 1°44′35.9″ E at 400 m altitude and 87 km from Barcelona. More information about the orchard and the environmental and agronomical conditions are detailed elsewhere [11]. Before the oil production, the olives were washed with water. The olives were crushed using a 5 mm sieve, and the water addition was 10 L/h. The EVOOs were produced in an industrial mill (OLIOMIO 200 PROFY, MORI-TEM) by the company MIGJORN (Valls de Torroella, Catalonia, Spain) on two consecutive days, 13 and 14 October 2021, and kindly provided to our research group by the same company. The tested variables were temperature (18 and 23 °C) and time (30, 40 and 50 min) of malaxation.

Six different EVOOs were produced with the same olive sample on the two days. O1, O2 and O3 were produced on 13 October and O4, O5, and O6 were produced the following day. The olives used for the elaboration of O4–O6 were stored in a tractor trailer at ambient temperature (from 14 to 21 °C) for 17 h overnight. To check whether olive storage could have altered the results, O4 was produced using the same malaxation conditions as O1. The EVOO samples were stored at −20 °C until the chemical analyses.

### 2.3. Physical Characterization of the Olives

The physical characterization of olives was carried out by the IRTA (Mas Bové) on the same day as the EVOO production, i.e., the characterization was performed twice, on 13 and 14 October. The RI was evaluated following the methodology described in Olmo-Cunillera et al. [11]. The weight of the olives was measured by gravimetric analysis. Additionally, a visual inspection was carried out to determine the condition of the olive samples.

### 2.4. Phenolic Extraction and Profiling

The phenolic compounds underwent liquid–liquid extraction as described in Olmo-Cunillera et al. [12]. The quantification was carried out by liquid chromatography coupled to mass spectrometry in tandem mode (LC-MS/MS) following the methodology also described in Olmo-Cunillera et al. [12]. An Acquity TM UPLC (Waters; Milford, MA, USA) coupled to an API 3000 triple-quadrupole mass spectrometer (PE Sciex, Concord, ON, Canada) with a turbo ion spray source was used. The column and precolumn were an Acquity UPLC^®^ BEH C18 column (2.1 mm × 50 mm, i.d., 1.7 µm particle size) and Acquity UPLC^®^ BEH C18 Pre-Column (2.1 mm × 5 mm, i.d., 1.7 µm particle size) (Waters Corporation^®^, Wexford, Ireland), respectively.

The quantification was done with an external calibration curve using refined olive oil with the following standards: apigenin, hydroxytyrosol, *p*-coumaric acid, pinoresinol, oleuropein, ligstroside, oleocanthal, oleacein, oleuropein aglycone, and elenolic acid. The concentrations employed for all standards were 0, 1, 2, 5, 8, 10, and 20 ppm. The refined olive oils with the standards underwent the same liquid–liquid extraction as the EVOO samples. Compounds without a corresponding commercial standard were quantified using a phenolic standard with a similar chemical structure.

### 2.5. Fatty Acid Extraction and Profiling

FAs were extracted using the method for FA methyl esters (FAME) described in Olmo-Cunillera et al. [13] with a few modifications. A total of 25 mg of oil was weighed in a 10 mL tube and 40 µL of the internal standard (methyl tridecanoate, C13) was added at 1000 mg/L. Firstly, after the addition of 2 mL of 0.5 N sodium methoxide, the solution was stirred for 30 s and immediately heated at 100 °C for 15 min. The samples were then cooled in an ice bath. Secondly, 2 mL of 14% boron trifluoride was added to the samples, and the solution was again stirred for 30 s and heated at 100 °C for 15 min, before cooling in an ice bath. Thirdly, 1 mL of hexane was added to the samples, and the solution was stirred for 1 min. After the incorporation of 2 mL of saturated NaCl, the samples were stirred again for 30 s. Finally, the samples were centrifuged at 3000 rpm for 7 min, and 250 µL of the hexane phase was collected with a micropipette and stored in vials at −20 °C until analyzed.

Fast GC analyses were performed on a Shimadzu GC-2010 Gas Chromatograph (Shimadzu, Kyoto, Japan) equipped with a flame ionization detector and a Shimadzu AOC-20i Autoinjector. Separation of fatty acid methyl esters was carried out on a capillary column (40 cm × 0.18 mm i.d. 0.1 µm film thickness) coated with an RTX-2330 stationary phase of 10% cyanopropyl phenyl—90% biscyanopropyl polysiloxane from Restek (Bellefonte, PA, USA). Operating conditions are described in Olmo-Cunillera et al. [13].

The concentration of each FA was calculated considering the area and concentration of the internal standard, applying the following equation,

(A_i_ × C_IS_)/(A_IS_ × M_S_), (1)

where A_i_ is the area of the FA; C_IS_, the concentration of the internal standard; A_IS_, the area of the internal standard; and M_S_, the mass of the sample. The percentage of composition was calculated by dividing the area of the FA between the area of the sum of all identified FAs and multiplying by 100.

### 2.6. Determination of Carotenoids, Chlorophylls, α-Tocopherol, and Squalene

The determination of the carotenoids (lutein and *β*-carotene), chlorophylls, *α*-tocopherol (vitamin E), and squalene was done with a 200:800 (*v*/*v*) (EVOO:TBME) dilution in amber vials and performed by LC [12]. An Acquity TM UPLC coupled to a photodiode detector (PDA) (Waters Corporation^®^; Milford, MA, USA) was used. The column was a YMCTM C30 (250 mm × 4.6 mm, i.d., 5 µm particle size) (Waters Corporation^®^, Milford, MA, USA). The mobile phases were TBME:methanol (8:2 *v*/*v*) (A) and methanol (B). An increasing linear gradient (*v*/*v*) of A was used (t (min), %A) as follows: (0, 10); (10, 25); (20, 50); (25, 70); (35, 90); (43, 94); (45, 19); (55, 10). The method had a constant flow rate of 0.6 mL/min, and an injection volume of 10 µL. The absorbance was measured at 450 nm for carotenoids (lutein and *β*-carotene) and at 210 nm for *α*-tocopherol and squalene.

For the quantification of each compound, an external calibration curve of the corresponding commercial standard was employed (lutein, *β*-carotene, chlorophyll a, *α*-tocopherol, and squalene). The following concentrations were employed: 0.1, 0.5, 1, 2, 5, and 10 ppm for chlorophyll a, lutein, and *β*-carotene; 2, 5, 10, 15, 20, and 30 ppm for *α*-tocopherol, and 20, 50, 75, 100, 150, and 200 for squalene.

### 2.7. Extraction and Determination of the Antioxidant Capacity (DPPH Free Radical Scavenging Assay) and Oxidative Stability (Rancimat)

The extraction method for the DPPH assay was as follows. A sample of 0.5 g of EVOO was dissolved in 1 mL of hexane in a 10 mL centrifuge tube and shaken for 30 s. A total of 2 mL of methanol:H_2_O (8:2) was added, and the samples were shaken again for 30 s. Afterwards, the two phases were separated using centrifugation at 3000 rpm and 4 °C for 4 min. The methanolic fraction was collected in another centrifuge tube and underwent a second cleaning with 1 mL of hexane, whereas the hexane fraction was again treated with 2 mL of methanol:H_2_O (8:2) to recover the remaining phenolic compounds. All tubes were shaken for 30 s and centrifuged at 3000 rpm and 4 °C for 4 min. The methanolic phases were recovered together and stored at −20 °C until the TPC and DPPH analysis.

The DPPH radical scavenging activity assay was performed based on the reduction of the DPPH• radical by antioxidants, as described in Olmo-Cunillera et al. [11]. Results were expressed as µg of Trolox equivalents (TE) per g of oil for DPPH. Trolox was used as the standard to prepare a calibration curve for DPPH (linearity range: 5–100 µg/mL, R^2^ > 0.927).

The oxidative stability was evaluated with the Rancimat method [14]. This technique measures the oxidative stability of oils and fats in accelerated conditions and is based on the induction of sample oxidation by exposure to high temperatures and air flow. Therefore, the longer the induction time, the more stable the sample. A mass of 3 g of EVOO sample was heated at 120 °C with a constant air flow of 20 L/h. The results were expressed as the induction time of oxidation (in hours), measured with the Rancimat 743 apparatus (Metrohm Co., Basilea, Switzerland). The induction time of oxidation is the time required to cause a sudden change in the conductivity of an aqueous solution where the volatile compounds resulting from the oil oxidation are collected.

### 2.8. Statistical Analysis and Multivariate Analysis

All the analyses were done in triplicate. Statgraphics Centurion 18 software, version 18.1.13 and RStudio, version 2022.12.0 Build 353 (R Project for Statistical Computing version 4.2.2) were used to perform the analysis of variance. First, the normality of data and the homogeneity of variance were tested by the Shapiro–Wilk test and Levene’s test, respectively. An analysis of variance of two factors (two-way ANOVA) with a Tukey test was applied to evaluate the effect of the malaxation conditions on the oil samples O1, O2, O3, O5, and O6 when the assumptions of normality and homogeneity of variance were met (*p* ≥ 0.05). If any of these assumptions were not met (*p* < 0.05), a nonparametric statistical test was applied (Kruskal–Wallis with a pairwise Mann–Whitney U as a post hoc test). To evaluate the effect of the olive storage time in the tractor trailer on the EVOO samples O1 and O4, a one-way ANOVA with Tukey HSD test was used when the assumptions of normality and homogeneity of variance were met (*p* ≥ 0.05). If any of these assumptions were not met (*p* < 0.05), a nonparametric statistical test was applied (Kruskal–Wallis with Bonferroni correction). In addition, a two-way ANOVA was performed to determine possible interactions between the malaxation factors (temperature and time).

For the multivariate analysis, the software used was SIMCA 13.0.3.0 (Umetrics, Umeå, Sweden). All the composition data (content of phenolic compounds, Fas, carotenoids, chlorophylls, *α*-tocopherol, and squalene) as well as the Rancimat and DPPH data were included. An unsupervised approach, specifically a principal component analysis (PCA), was performed. The data were standardized with UV scaling and mean centering.

## 3. Results and Discussion

### 3.1. Physical Characterization of the Olives

The olive samples used to produce EVOO on either of the two days of production had very similar physical characteristics. The RI of the olives processed on 13 and 14 October was 1.14 ± 0.11 and 1.20 ± 0.05, and the weight 1.83 ± 0.23 g and 1.80 ± 0.17 g, respectively. Overall, all the samples were in good condition, although some olives had suffered minor damage due to the harvesting machine employed. The damage was a bit more noticeable after 17 h of storage.

### 3.2. Effect of Olive Storage on EVOO Composition and Oxidative Stability

The EVOO samples O1 and O4 were produced under the same malaxation conditions (18 °C and 30 min) but on different days. O1 was produced on the same day the olives were harvested and O4 the following day, after the olives had been stored for 17 h overnight in a tractor trailer at ambient temperature.

**Figure 1:**
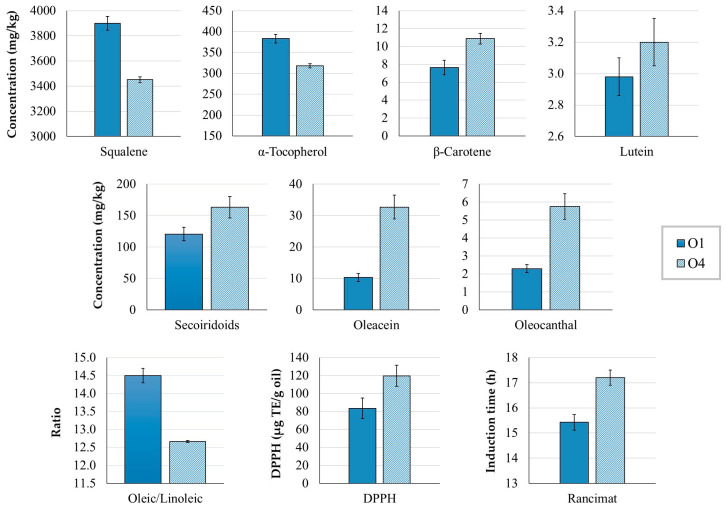
Concentration (mg/kg oil) of squalene, *α*-tocopherol, *β*-carotene, lutein, secoiridoids, oleacein, and oleocanthal in the EVOO samples O1 and O4, as well as the oleic/linoleic ratio, antioxidant capacity by DPPH (µg TE/g oil), and oxidative stability by Rancimat (induction time (h)). O1 was produced on the day the olives were harvested, and O4 on the day after harvesting with stored olives. Both EVOOs were malaxed at 18 °C for 30 min. Results are expressed as mean ± standard deviation, *n* = 9. All variables differed significantly (*p* < 0.05) between samples.

The olive storage had a negative effect on the content of *α*-tocopherol and squalene (Figure 1), a positive effect on the secoiridoid content, and no effect on the total Fas (Table S1), in agreement with a previous report [10]. These changes can be expected, as olive storage enhances the activity of hydrolytic and oxidative enzymes [15]. Additionally, carotenoids (lutein and *β*-carotene) increased (Figure 1), whereas chlorophyll levels were unaltered (Table 1).

The sum of phenolic compounds was not significantly affected by extracting the oil a day after the olive harvest, even though it was slightly higher in O4 (Table 1). However, most of the individual phenolic compounds decreased significantly, most likely due to the action of oxidative enzymes such as polyphenol oxidase (PPO) and peroxidase (POX). When olives are damaged, the oxygen required for the oxidoreductase reactions can enter the fruit, which also favors the proliferation of microorganisms such as yeasts and bacteria, another possible factor contributing to the phenolic loss [15]. In contrast, secoiridoid levels increased, particularly oleuropein aglycone, oleacein, and oleocanthal (Figure 1). This behavior can be attributed to the action of hydrolytic enzymes such as *β*-glucosidase and esterases during the 17 h of storage. Another relevant factor is that plant synthesis of phenolic compounds is activated as a defense response to repair damage [16]. For example, oleuropein aglycone has been associated with a response to wounding stress in olives [17]. The decrease in *α*-tocopherol and squalene could also be due to oxidative reactions [18]. In addition, the activity of enzymes involved in sterol biosynthesis could contribute to the depletion of squalene [19]. 

Olive storage affected the content of carotenoids, which increased, whereas chlorophyll levels decreased, even though it was not statistically different. Chlorophyll is susceptible to photooxidation, but this process was limited as the 17 h of storage was mainly at night, which could also explain why carotenoids, strong protectors against photosensitized oxidation [5], were not depleted. Additionally, *α*-tocopherol can contribute to the protective effect of carotenoids, avoiding their loss [20]. The increase in carotenoids in the EVOO could be attributed to the degradation of chloroplast membranes during olive storage, which enhances extractability during malaxation [21]. 

Finally, while olive storage did not alter the total FA content, some individual Fas were affected (Table S1). C15:0, C15:1, and linoleic (C18:2 n-6) acids increased, whereas C20:2 n-6, C22:0, C22:1 n-9, C22:2 n-6, C23:0, and C24:0 decreased. Therefore, the very-long-chain Fas (more than 18C) seem to have been damaged by olive storage. Possible explanations could be related to the inactivation of the elongases involved in their biosynthesis [22], or to FA degradation over time. The activity of specific desaturases has been associated with an increase of linoleic acid [23], which in the present study resulted in a significant reduction of the oleic/linoleic and MUFA/PUFA ratios (Figure 1), an indicator that the oil has lost oxidative stability.

However, despite having a lower oleic/linoleic ratio and a reduced concentration of *α*-tocopherol and squalene, O4 had significantly higher DPPH and Rancimat values (Table 1, Figure 1). These findings reflect that phenolic compounds, especially the secoiridoids oleacein, oleocanthal, and oleuropein aglycone, contributed strongly to both the antioxidant capacity and oxidative stability of the oil. The high antioxidant capacity of secoiridoids, especially *o*-diphenols, has been reported previously [4,24]. In other olive cultivars, Rancimat values have been found to remain unaltered over several days of storage [15]. In the case of ‘Corbella’ olives, our results show that storing healthy fruit with an RI of 1 to 1.5 for 17 h overnight before EVOO production enhances the oxidative stability of the oil.

### 3.3. Effect of Malaxation Conditions on the EVOO Composition and Oxidative Stability

#### 3.3.1. Phenolic Compounds

Malaxation conditions had variable effects on the different phenolic compounds (Table 1). Although the sum of phenolic compounds was not altered by malaxation, phenolic alcohols and flavonoids were negatively affected by the higher temperature (*p* < 0.05) and showed no significant effects due to malaxation time. The higher temperature also negatively affected the secoiridoids, as previously reported [7,12,25,26], but their content increased with malaxation time. 

Among the secoiridoids, which are the major group of phenolic compounds in olive oil, oleuropein aglycone is predominant in ‘Corbella’ olives and EVOOs [7,11]. The effect of the duration of malaxation on secoiridoids differed with the temperature. At 18 °C the levels of oleuropein aglycone increased slightly with time, whereas at 23 °C they decreased slightly. Similar tendencies were observed for ligstroside aglycone but without significant differences. Both oleocanthal and oleacein increased with time and temperature, as found in the pilot study [7]. Finally, hydroxyelenolic acid, oleocanthalic acid, and hydroxyoleuropein aglycone, which are oxidized derivatives of secoiridoids [27,28], showed significant differences only in O1 malaxed at 18 °C for 30 min, when their concentration was highest. Although elenolic acid is not a phenolic compound, it forms part of the chemical structure of secoiridoids [29] and is generated by their degradation [30]. An increase in both temperature and time of malaxation had a negative effect on the EVOO elenolic acid content, as previously reported [7]. ‘Corbella’ olives are characterized by a high content of this compound [11].

The high concentration of oleuropein aglycone and elenolic acid in ‘Corbella’ olives suggests this cultivar has a high *β*-glucosidase activity [30]. Although oleacein and oleocanthal increased with malaxation temperature, presumably due to esterase activity [31], their levels remained low. This indicated that the tested conditions were not optimal for the activity of these enzymes, which is reported to be enhanced at 30 °C [7,31]. Likewise, longer malaxation times significantly increased oleacein and oleocanthal content, as the esterases had more time to develop their activity. Additionally, the difference in oleacein and oleocanthal levels corresponded to the concentration of their precursors, the considerably higher concentration of oleuropein aglycone compared to ligstroside aglycone explaining the higher formation of oleacein versus oleocanthal. The fact that the levels of both aglycones were similar or differed only slightly in the EVOO samples suggests their catabolic and anabolic pathways were balanced. Thus, as well as being transformed by esterases to oleacein and oleocanthal, the aglycones could have been formed from oleuropein and ligstroside by *β*-glucosidase activity [30]. Three products of secoiridoid oxidation were found, hydroxyelenolic acid, oleocanthalic acid, and hydroxyoleuropein aglycone. Their low and generally constant concentration in all the EVOO samples indicates this oxidation process was not very active. The content of hydroxyelenolic acid was highest and that of oleocanthalic acid lowest, which corresponds with the levels of their respective precursors, elenolic acid and oleocanthal.

Two phenomena can contribute to the depletion of phenolic compounds during malaxation: the activity of oxidative and hydrolytic enzymes [32], and the transfer of hydrophilic phenols to the water phase [33]. Both phenomena increase with longer malaxation times. According to our results, as the oxidative products did not increase with malaxation time, it seems more likely that the depletion of elenolic acid could be attributed to its transfer to the water phase. This is supported by the observation that hydroxytyrosol, also a degradation product of secoiridoids, did not increase with malaxation temperature or time. Additionally, hydroxytyrosol levels were only significantly lower at 23 °C and 40 min, suggesting that its degradation or transfer to the water phase can occur in these malaxation conditions.

The flavonoids apigenin and luteolin were negatively affected by increasing the temperature of malaxation, as reported in other studies [7], whereas a longer malaxation time reduced their content only at 18 °C. The same behavior was observed for hydroxytyrosol acetate and the lignan pinoresinol, which were depleted when the malaxation time was increased at 18 °C. Finally, the levels of *p*-coumaric acid decreased when both malaxation parameters were increased, indicating a susceptibility to degradation or transfer to the water phase.

According to these results, malaxation at 18 °C for 30 min provides the most favorable conditions to obtain ‘Corbella’ EVOO with high concentrations of phenolic compounds. However, if the goal is also to obtain EVOOs with a high content of oleocanthal and oleacein, malaxation should be applied at 18 °C for 50 min, as their concentration is enhanced by higher temperatures or longer times.

#### 3.3.2. Fatty Acid Profile

The FA profile was the same in all EVOO samples, regardless of the malaxation conditions applied (Table S2). The main FA was oleic acid (C18:1 n-9) (77.75–78.89%), followed by palmitic acid (C16:0) (11.68–11.86%), linoleic acid (C18:2 n-6) (5.44–6.69), stearic acid (C18:0) (1.78–1.90%), 9-palmitoleic acid (C16:1 n-7) (0.59–0.64), *α*-linolenic acid (C18:3 n-3) (0.52–0.57%), arachidic acid (C20:0) (0.28–0.29%), gondoic acid (C20:1 n-9) (0.20–0.22%), and behenic acid (C22:0) (0.08–0.10%). The percentage of the other Fas was <0.10%. The FA composition (%) of the samples (Table S2) fell within the limits established for EVOO by the European Commission No 2022/2104 [34] and coincides with the FA profile previously reported for ‘Corbella’ olives [11].

‘Corbella’ EVOO has a higher proportion of oleic acid, and less palmitic, linoleic, 9-palmitoleic, arachidic, and gondoic acids than ‘Arbequina’ EVOO [13], and more palmitic and less oleic, stearic, linoleic, *α*-linolenic, and arachidic acids than ‘Picual’ EVOO [35]. Variations in the FA composition of olive oils of different cultivars are due to genetic differences [2], such as the variable capacity or expression of desaturase enzymes involved in FA biosynthesis [23]. 

The total FA content was not significantly affected by any of the factors studied, with values ranging between 817.80 mg/g and 866.36 mg/g in all the EVOO samples (Table S1), although it tended to increase with the malaxation temperature. At higher temperatures, viscosity is reduced, and coalescence of oil droplets is enhanced, so the oily phase becomes richer in oil and poorer in other compounds, especially unsaponifiable lipids and water [13].

The most abundant Fas, oleic and palmitic acids, did not show any significant differences between samples. Nevertheless, the concentration of relevant Fas such as palmitoleic, linoleic, *α*-linolenic, and gondoic acids increased at the higher temperature, as reported in ‘Arbequina’ EVOOs [13]. Linoleic acid was affected by an interaction of both malaxation parameters. At 18 °C, its concentration tended to increase with malaxation time, whereas at 23 °C it tended to decrease, suggesting that prolonging the malaxation at high temperatures promoted its oxidation or lipoxygenase activity [36].

Increasing both malaxation parameters reduced the MUFA/PUFA and oleic/linoleic ratios, indicating that higher temperatures and longer times of malaxation produce EVOOs more susceptible to oxidation processes. Accordingly, the most stable EVOO was produced by malaxation at 18 °C for 30 min (MUFA/PUFA = 13.21 ± 0.17, oleic/linoleic = 14.50 ± 0.20), followed by 18 °C for 40 min (MUFA/PUFA = 12.79 ± 0.05, oleic/linoleic = 13.93 ± 0.05) (Table S1).

A previous analysis of ‘Corbella’ olives with an RI similar to that of the olives used in the present study found lower values for the two ratios [11] compared to the ‘Corbella’ EVOOs, indicating the oxidative stability was enhanced during the production process. Hernández et al. [2] compiled a list of the oleic/linoleic ratios of olive oils produced from 89 cultivars from the Worldwide Olive Germplasm Bank of Cordoba. According to these values, ‘Corbella’ EVOOs would be ranked between 10^th^ and 15^th^. However, the ratios of that study were obtained from EVOOs produced with olives harvested 28–31 weeks after flowering, i.e., with an RI above 2.

An ‘Arbequina’ EVOO produced from olives with an RI between 1.16 and 2.26 and using different malaxation conditions [13] had an oleic/linoleic ratio between 6.21 and 7.82, which is considerably lower than the ratio of ‘Corbella’ EVOOs (11.62–14.50). The ‘Arbequina’ ratio reported by Hernández et al. [2] was even lower (4.17). Linoleic acid is generated by the desaturation of oleic acid, and in some olive cultivars, such as ‘Picual’, ‘Arbequina’, and ‘Picudo’, the content of this PUFA increases with maturation due to a high expression of desaturase genes [23], resulting in a decrease in the oleic/linoleic ratio. However, in ‘Corbella’ olives the ratio was found to increase with ripeness up to an RI of 2 [11], suggesting this cultivar has a different expression pattern of the desaturases involved in the biosynthesis of both FA. Considering these results, it is likely that ‘Corbella’ EVOOs produced from olives with an RI of 2 would have a higher oleic/linoleic ratio, and would therefore be more stable than cultivars with a higher linoleic acid content, such as ‘Arbequina’. As mentioned, the oleic/linoleic ratio differs between ‘Corbella’ and ‘Arbequina’ EVOOs because the former has a higher proportion of oleic acid and lower proportion of linoleic acid. Accordingly, ‘Corbella’ olives seem to be a suitable choice for the production of EVOOs with high oleic/linoleic ratios. However, before reaching a definitive conclusion, the evolution of the ratio should be tracked over the whole maturation process of ‘Corbella’ olives.

#### 3.3.3. Carotenoids, Chlorophylls, α-Tocopherol, and Squalene

All the pigments (lutein, *β*-carotene, and chlorophylls) increased with longer malaxation (Table 1), because, as previously reported, there was more time for their transfer to the oily phase [12,37]. However, chlorophylls decreased at the higher temperature. Pigments are susceptible to degradation when exposed to temperature and oxygen X [5,38,39,40]. Therefore, the balance between the transfer and the degradation determines the final pigment content in the oil. Furthermore, it was previously reported that the loss caused by the oil extraction process is more marked for the chlorophylls than for the carotenoids [41,42], suggesting that chlorophylls could be more susceptible to degradation than carotenoids.

*Α*-Tocopherol and squalene were negatively affected by the higher malaxation temperature and times; a decrease in levels due to a higher temperature has been reported in other studies [12,43]. Tocopherols are strong antioxidants that protect olive oil from lipid oxidation [1], so an oxidation process during malaxation could have caused their depletion in our study. Squalene also has a protective effect, helping to prevent the temperature-dependent autoxidation of PUFAs [44]. Additionally, as an unsaturated molecule, squalene is unstable and easily oxidized, which could also explain the depletion observed [19]. As previously discussed, the PUFA content increased slightly with the malaxation temperature. Rastrelli et al. [18] found that PUFA levels remained constant during 8 months of EVOO storage, and only started to decline when antioxidant levels had decreased considerably. Therefore, the decrease in *α*-tocopherol and squalene in the EVOO samples could be related to their contribution to protecting PUFAs from thermal oxidation.

#### 3.3.4. Oxidative Stability (Rancimat) and Antioxidant Capacity (DPPH Assay) of the EVOO Samples

Increasing the temperature without changing the malaxation time led to a slight increase in the oxidative stability of the EVOO samples (Table 1). The same pattern was observed when the malaxation time was extended without altering the temperature. The EVOO with the highest oxidative stability was produced by malaxation at 18 °C for 50 min.

When the temperature was increased without changing the malaxation time, the DPPH assay revealed that the resulting EVOOs had a higher antioxidant capacity (Table 1). In correlation with the results for optimum oxidative stability, the best values were obtained with conditions of 18 °C/50 min.

The increase in antioxidant activity correlates with the higher levels of the strongly antioxidant phenolics hydroxytyrosol, oleuropein aglycone, oleocanthal and oleacein, as well as the carotenoids lutein and *β*-carotene. A high contribution of phenolic compounds, especially *o*-diphenols, together with carotenoids, to the oxidative stability measured by Rancimat has been previously reported [1,45]. Thus, in agreement with the results obtained when analyzing the effect of olive storage, the highest antioxidant capacity and oxidative stability were observed in EVOOs with the highest content of phenolic compounds, especially oleacein, oleocanthal, and oleuropein aglycone.

### 3.4. Principal Component Analysis

**Figure 2:**
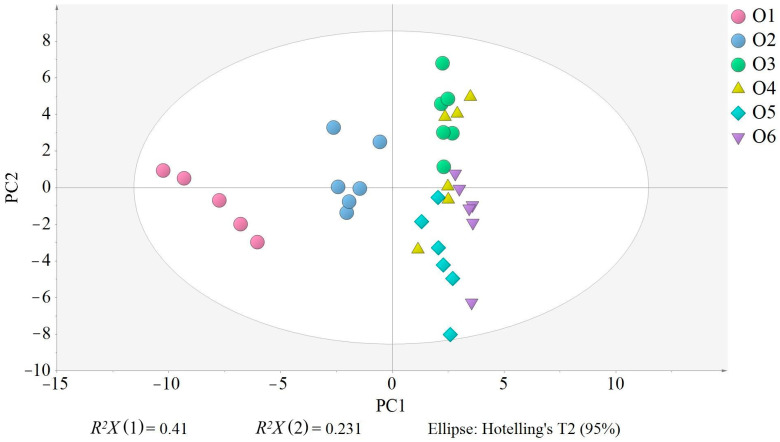
Score scatter plot of the principal component analysis (PCA). EVOO samples are colored and shaped according to their production conditions: O1 (no storage, 18 °C/30 min), O2 (no storage, 18 °C/40 min), O3 (no storage, 18 °C/50 min), O4 (17 h storage, 18 °C/30 min), O5 (17 h storage, 23 °C/30 min), and O6 (17 h storage, 23 °C/40 min). *R*^2^*X* (1) and *R*^2^*X* (2) in the PCA are the variations explained by the first PC and the second PC, respectively, together explaining 66.3% of the variation. All samples were inside the Ellipse Hotelling’s T2, indicating there were no strong outliers.

**Figure 3:**
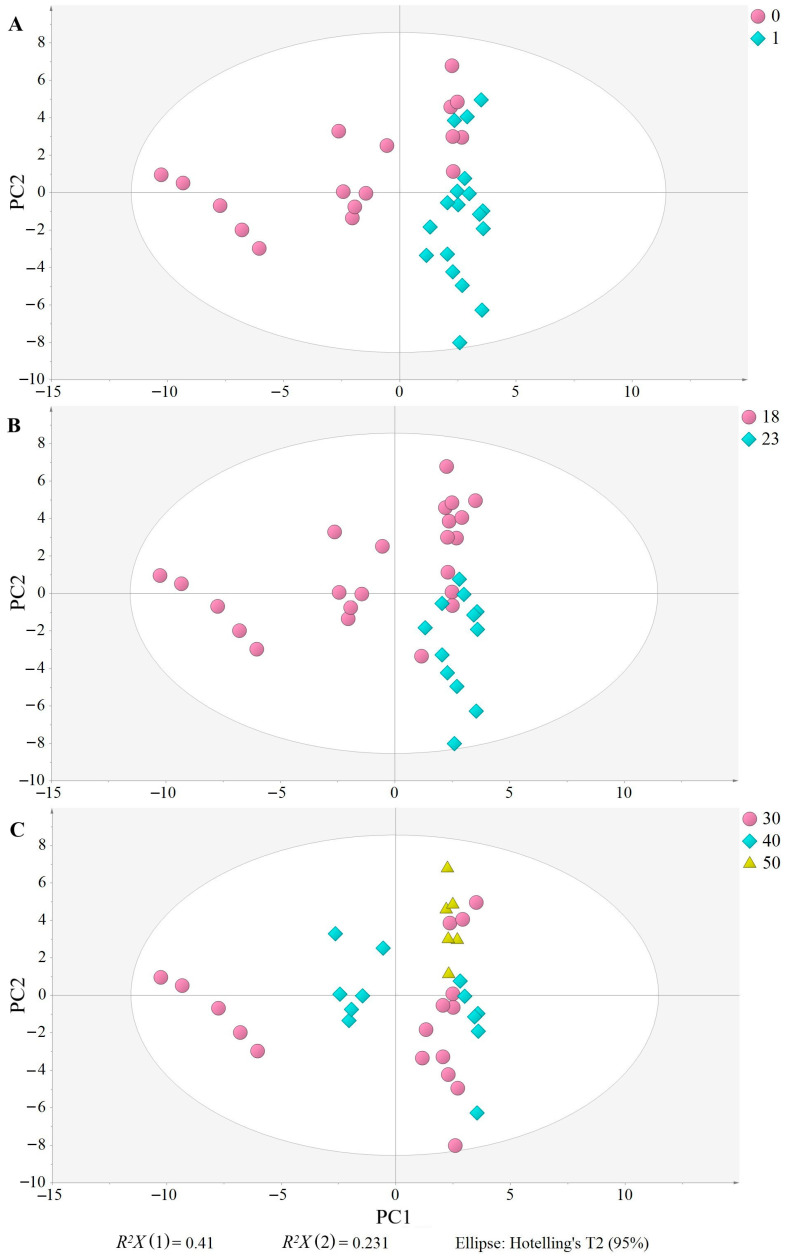
Score scatter plot of the principal component analysis (PCA). EVOO samples are colored and shaped according to the olive storage (**A**) (0: no storage, 1: 17 h of storage), (**B**) malaxation temperature (18 °C, 23 °C), and malaxation time (**C**) (30 min, 40 min, 50 min). *R*^2^*X* (1) and *R*^2^*X* (2) in the PCA are the variations explained by the first PC and the second PC, respectively, together explaining 66.3% of the variation. All samples were inside the Ellipse Hotelling’s T2, indicating there were no strong outliers.

The PCA model with five PC had an explained variation (*R*^2^*X*) of 0.848 and a predicted variation (*Q*^2^*X*) of 0.651. Two plots are basic to understand the PCA, the score plot and the loading plot, which show the relationships among the samples and variables, respectively. Thus, the closer the samples or variables, the more related. In the score plot (Figure 2), O1 (18 °C, 30 min) is clearly separated from the other samples and located on the left side, showing that the composition of O1 samples greatly differs from the others. O2 (18 °C, 40 min) is clustered in the middle of the plot, but closer to the remaining samples, also indicating a difference in composition but not as great as O1. Finally, the other samples (O3, O4, O5 and O6) are on the right side of the plot, and their proximity indicates a more similar composition. Although all three factors evaluated (malaxation temperature and time, and olive storage) seem to contribute to the separation of the samples (Figure 3A–C), olive storage appears to be the most influential, as samples produced on the day of harvesting are distributed on the left side, appearing on the right side when produced the following day (Figure 3A). O3 samples are an exception, as they appear on the right side of the plot, despite being produced on the day of harvesting, indicating that the malaxation conditions (18 °C, 50 min) resulted in EVOOs with a similar composition to those produced with stored olives. Nevertheless, O3 samples are positioned toward the upper right of the plot, similar to O4, while O5 and O6 are more in the bottom right, indicating that the malaxation conditions still have an influence on the separation.

**Figure 4:**
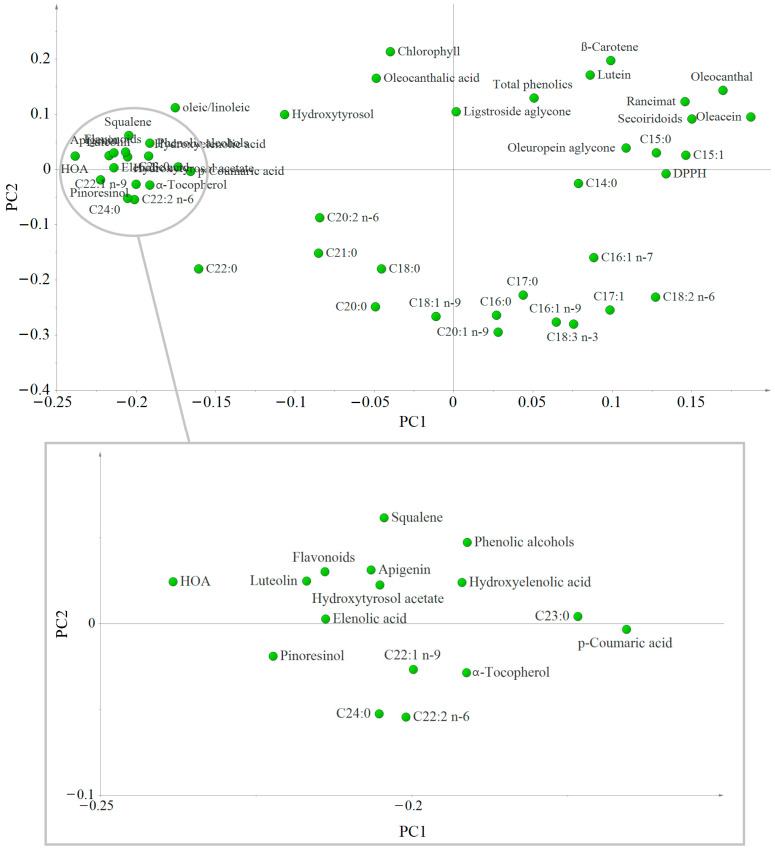
Loading scatter plot of the first and second principal components of the PCA showing the distribution and correlation of the different variables analyzed in the ‘Corbella’ EVOO samples. The variables located far from the plot origin correlate to the samples positioned in the same part of the scatter plot (see Figure 2). HOA: Hydroxyoleuropein aglycone; HTA: Hydroxytyrosol acetate; HEA: Hydroxyelenolic acid.

To interpret the distribution seen in the score plot, the loading plot was performed (Figure 4). The variables located far from the plot origin correlate to the samples positioned in the same part of the scatter plot (Figure 2). Thus, the variables most associated with O1 samples are the majority of the phenolic compounds (except the secoiridoids), *α*-tocopherol, squalene, the oleic/linoleic ratio, and the very-long-chain Fas (C22:0, C22:1 n-9, C22:2 n-6, C23:0, and C24:0), and that the samples produced the day after harvesting (right side of the plot) had a higher content of the other Fas and secoiridoids. O2 samples are associated with the same variables as O1, but to a lesser extent, because the separation between samples and variables is greater in this case. Additionally, the loading plot gives information about the relationships among the variables. The proximity of Rancimat values to secoiridoids, particularly oleacein and oleocanthal, corroborates the strong positive correlation between these variables. DPPH values and oleuropein aglycone are also situated quite closely to these variables, as are lutein and *β*-carotene, indicating a positive correlation. These positive correlations demonstrate the contribution of these compounds to the oil oxidative stability: the closer to Rancimat, the greater the contribution. Therefore, secoiridoids, especially oleacein and oleocanthal, are the major contributors. Benito et al. [41] also found a very good correlation between oleacein and total secoiridoids and oxidative stability of ‘Arbequina’ EVOOs. In addition, a possible synergistic effect between secoiridoids and carotenoids could enhance the antioxidant activity, as also envisaged by previous studies [45,46]. All these variables are associated with O3 and O4 samples, as their position in the loading and scatter plot match (upper right side).

## 4. Conclusions

This study of ‘Corbella’ EVOO, which was aimed at improving its oxidative stability, revealed two significant conclusions. First, linoleic acid was favored by olive storage and a higher malaxation temperature. Consequently, the oleic/linoleic ratio was higher at the lower malaxation temperature and time (18 °C and 30 min), and when the oil was produced on the same day of olive harvest. Accordingly, the ‘Corbella’ cultivar seems to be a promising candidate for the production of EVOOs with a high oleic/linoleic ratio. Second, although producing the EVOOs on the day of the olive harvest with malaxation at 18 °C for 30 min resulted in a better composition in terms of *α*-tocopherol, squalene, and oleic/linoleic ratio, these conditions did not produce the best values of antioxidant activity and oxidative stability. In fact, the EVOOs with the optimum antioxidant capacity and oxidative stability were obtained by malaxating at the higher temperature and times, and after storing the olives overnight. These desirable attributes were positively correlated with the content of secoiridoids, especially oleacein and oleocanthal. A synergistic effect between these two secoiridoids and carotenoids should not be discarded.

The results of this study therefore indicate that secoiridoids contribute strongly to the antioxidant capacity and oxidative stability of ‘Corbella’ EVOOs, and that oils with a high content of oleacein and oleocanthal will be more stable and have a longer shelf life. According to this study, storing the olives at environmental temperature overnight and performing the malaxation at least at 23 °C for 40–50 min (depending on the temperature), will increase the oleacein and oleocanthal content and thus the oxidative stability of EVOOs. These findings signify a notable advancement and hold substantial utility and significance in addressing and enhancing EVOO stability.

Future research should be focused on how the content of oleacein and oleocanthal can be even more enhanced by studying the factors involved in their accumulation, such as agronomic and climatic conditions, fruit ripeness, and technological aspects of oil extraction. Furthermore, an evaluation of EVOO quality and stability during long storage, as well as interventional studies would be of great relevance to see the impact of these two secoiridoids on EVOO shelf life and human health.

## Figures and Tables

**Table 1:** Concentration of phenolic compounds (mg/kg), carotenoids (lutein and *β*-carotene), chlorophylls, *α*-tocopherol (vitamin E), and squalene (mg/kg), and antioxidant capacity (DPPH) (µmg TE/g olive fruit), and oxidative stability (Rancimat (h)) of the EVOO samples. All results are expressed as mean ± standard deviation, *n* = 9. Different letters mean significant differences (*p* < 0.05) between samples for every variable (row), with increasing letters indicating increasing values. Letters are used for the malaxation study, and Greek letters for the olive storage study.

Sample ID	O1	O2	O3	O4	O5	O6
Production date	13 October 2021	13 October 2021	13 October 2021	14 October 2021	14 October 2021	14 October 2021
Malaxation temperature (°C)	18	18	18	18	23	23
Malaxation time (min)	30	40	50	30	30	40
**Phenolic compounds (mg/kg)**						
Sum of phenolics	165.90 ± 18.31 ^a,α^	163.81 ± 16.06 ^a^	191.21 ± 9.11 ^a^	180.87 ± 17.26 ^α^	174.77 ± 25.67 ^a^	169.10 ± 3.50 ^a^
*Secoiridoids*	120.68 ± 10.80 ^a,α^	134.97 ± 18.74 ^ab^	170.82 ± 7.49 ^c^	163.40 ± 16.91 ^β^	157.60 ± 23.08 ^bc^	152.82 ± 3.69 ^bc^
Ligstroside aglycone	11.83 ± 1.72 ^a,α^	12.94 ± 1.89 ^a^	13.45 ± 1.51 ^a^	12.60 ± 1.57 ^α^	12.01 ± 1.56 ^a^	11.50 ± 0.75 ^a^
Oleuropein aglycone	82.72 ± 7.47 ^a,α^	87.52 ± 12.49 ^ab^	105.92 ± 8.59 ^bc^	103.75 ± 12.05 ^β^	109.39 ± 18.27 ^c^	91.59 ± 3.84 ^abc^
Oleocanthal	2.30 ± 0.22 ^a,α^	3.92 ± 0.33 ^b^	6.90 ± 0.37 ^d^	5.76 ± 0.71 ^β^	3.97 ± 0.22 ^b^	5.64 ± 0.47 ^c^
Oleacein	10.31 ± 1.23 ^a,α^	16.14 ± 1.51 ^b^	36.05 ± 2.95 ^d^	32.72 ± 3.78 ^β^	23.22 ± 2.21 ^c^	35.53 ± 2.12 ^d^
Hydroxyelenolic acid	9.72 ± 1.03 ^b,β^	5.70 ± 0.48 ^a^	5.54 ± 0.76 ^a^	5.34 ± 0.77 ^α^	5.37 ± 0.62 ^a^	5.38 ± 1.08 ^a^
Oleocanthalic acid	1.18 ± 0.07 ^b,α^	0.94 ± 0.09 ^a^	1.18 ± 0.09 ^b^	1.15 ± 0.08 ^α^	0.93 ± 0.07 ^a^	1.08 ± 0.08 ^ab^
Hydroxyoleuropein aglycone	2.90 ± 0.26 ^c,β^	1.98 ± 0.17 ^a^	1.74 ± 0.05 ^a^	1.76 ± 0.13 ^α^	1.86 ± 0.12 ^a^	1.77 ± 0.18 ^a^
*Secoiridoid derivatives*						
Elenolic acid *	552.70 ± 48.29 ^d,β^	298.86 ± 35.16 ^c^	225.64 ± 25.36 ^ab^	205.00 ± 13.83 ^α^	275.12 ± 20.61 ^bc^	195.50 ± 28.83 ^a^
*Phenolic alcohols*	5.96 ± 0.73 ^c,β^	4.96 ± 0.26 ^b^	4.62 ± 0.45 ^b^	3.68 ± 0.67 ^α^	4.13 ± 0.53 ^ab^	3.33 ± 0.24 ^a^
Hydroxytyrosol	2.97 ± 0.43 ^b,β^	2.38 ± 0.36 ^ab^	3.01 ± 0.37 ^b^	2.17 ± 0.35 ^α^	2.57 ± 0.36 ^b^	1.86 ± 0.19 ^a^
Hydroxytyrosol acetate	2.99 ± 0.32 ^b,β^	2.58 ± 0.32 ^b^	1.67 ± 0.21 ^a^	1.53 ± 0.22 ^α^	1.55 ± 0.18 ^a^	1.47 ± 0.08 ^a^
*Flavonoids*	3.78 ± 0.47 ^c,β^	3.15 ± 0.32 ^b^	2.60 ± 0.04 ^a^	2.49 ± 0.05 ^α^	2.49 ± 0.13 ^a^	2.45 ± 0.14 ^a^
Apigenin	2.43 ± 0.31 ^c,β^	2.01 ± 0.24 ^b^	1.49 ± 0.04 ^a^	1.39 ± 0.04 ^α^	1.37 ± 0.08 ^a^	1.37 ± 0.12 ^a^
Luteolin	1.45 ± 0.16 ^c,β^	1.23 ± 0.03 ^b^	1.11 ± 0.01 ^ab^	1.10 ± 0.03 ^α^	1.12 ± 0.06 ^ab^	1.08 ± 0.02 ^a^
*Phenolic acids*						
*p*-Coumaric acid	1.33 ± 0.03 ^c,β^	1.27 ± 0.02 ^b^	1.23 ± 0.03 ^ab^	1.27 ± 0.03 ^α^	1.28 ± 0.03 ^b^	1.21 ± 0.03 ^a^
*Lignans*						
Pinoresinol	29.52 ± 2.91 ^c,β^	19.25 ± 2.15 ^b^	11.94 ± 1.61 ^a^	9.85 ± 0.44 ^α^	9.46 ± 1.23 ^a^	8.08 ± 1.20 ^a^
DPPH (µg TE/g oil)	83.47 ± 11.66 ^ab,α^	77.20 ± 7.60 ^a^	114.63 ± 5.91 ^c^	119.81 ± 11.59 ^β^	117.08 ± 12.03 ^c^	102.02 ± 10.35 ^bc^
Rancimat (h)	15.43 ± 0.34 ^a,α^	15.83 ± 0.15 ^a^	18.72 ± 0.29 ^d^	17.20 ± 0.17 ^β^	16.39 ± 0.05 ^b^	16.97 ± 0.25 ^c^
**Carotenoids, chlorophylls, α-tocopherol, and squalene (mg/kg)**						
Lutein	2.98 ± 0.12 ^ab,α^	2.94 ± 0.12 ^a^	3.44 ± 0.16 ^c^	3.20 ± 0.15 ^β^	2.87 ± 0.13 ^a^	3.16 ± 0.13 ^b^
*β*-Carotene	7.66 ± 0.81 ^a,α^	9.55 ± 0.65 ^b^	12.02 ± 0.58 ^c^	10.88 ± 0.59 ^β^	7.08 ± 0.35 ^a^	9.92 ± 0.36 ^b^
Chlorophylls	3.51 ± 0.63 ^c,α^	4.24 ± 0.41 ^d^	5.50 ± 0.38 ^e^	3.06 ± 0.23 ^α^	1.77 ± 0.10 ^a^	2.62 ± 0.14 ^b^
*α*-Tocopherol	383.05 ± 10.51 ^b,β^	335.74 ± 11.03 ^a^	312.97 ± 4.95 ^a^	317.86 ± 5.05 ^α^	316.49 ± 31.73 ^a^	321.29 ± 5.59 ^a^
Squalene	3900.06 ± 54.48 ^d,β^	3555.40 ± 43.18 ^c^	3535.57 ± 41.82 ^c^	3451.40 ± 23.71 ^α^	3369.34 ± 63.20 ^a^	3444.33 ± 21.27 ^b^

* Elenolic acid was not included in the total phenolic content, as it is not a phenolic compound, but a degradation product.

## Data Availability

The data are not publicly available due to company’s privacy.

## Supplementary Materials

The following supporting information can be downloaded at: https://www.mdpi.com/article/10.3390/antiox12091776/s1, Table S1: Concentration of fatty acids (mg/g); Table S2: Fatty acid composition (%).
