# Supplementary material for: Oleacein and Oleocanthal: Key Metabolites in the Stability of Extra Virgin Olive Oil
Source: Antioxidants (Basel). 2023 Sep 18;12(9):1776. doi: 10.3390/antiox12091776 (PMC10525235; doi:10.3390/antiox12091776)
Supplement: Supplementary file 1 [file antioxidants-12-01776-s001.zip › antioxidants-2603420-supplementary.pdf]

SUPPLEMENTARY MATERIAL

Oleacein and oleocanthal: key metabolites in the stability of extra virgin olive oil

Alexandra Olmo-Cunillera [1,2], Maria Pérez [1,2], Anallely López-Yerena [1], Mohamed M. Abuhabib [1], Antònia Ninot [3], Agustí Romero-Aroca [3], Anna Vallverdú-Queralt [1,2], and Rosa Maria Lamuela-Raventós [1,2,*]

[1] Polyphenol Research Group, Department of Nutrition, Food Science and Gastronomy, Catalonia Food Innovation Network (XIA), Faculty of Pharmacy and Food Sciences, Institute of Nutrition and Food Safety (INSA-UB), University of Barcelona, 08028 Barcelona, Spain; alexandra.olmo@ub.edu; mariaperez@ub.edu; nayelopezye@ub.edu; mabuhaab8@alumnes.ub.edu; avallverdu@ub.edu

[2] CIBER Physiopathology of Obesity and Nutrition (CIBEROBN), Instituto de Salud Carlos III, 28029 Madrid, Spain.

[3] Institute of Agrifood Research and Technology (IRTA), Fruit Science Program, Olive Growing and Oil Technology Research Team, 43120 Constantí, Spain; antonia.ninot@irta.cat; agusti.romero@irta.cat

* Correspondence: lamuela@ub.edu

Table S1. Concentration of fatty acids (mg/g). Results are expressed as mean ± standard deviation, n = 9. Different letters mean significant differences ($p < 0.05$) between samples with increasing letters indicating increasing values. Letters are used for the malaxation study, and Greek letters for the olive storage study.

**Sample ID**	**O1**	**O2**	**O3**	**O4**	**O5**	**O6**
Production date	13/10/2021	13/10/2021	13/10/2021	14/10/2021	14/10/2021	14/10/2021
Malaxation temperature (°C)	18	18	18	18	23	23
Malaxation time (min)	30	40	50	30	30	40
FAs (mg/g)						
C14:0	0.16 ± 0.02 $^{a,\alpha}$	0.17 ± 0.02 ^{a}	0.17 ± 0.03 ^{a}	0.16 ± 0.02 $^{\alpha}$	0.17 ± 0.02 ^{a}	0.18 ± 0.02 ^{a}
C15:0	0.07 ± 0.01 $^{a,\alpha}$	0.08 ± 0.01 ^{a}	0.09 ± 0.02 ^{bc}	0.09 ± 0.01 $^{\beta}$	0.08 ± 0.01 ^{ab}	0.10 ± 0.02 ^{c}
C15:1	0.08 ± 0.01 $^{a,\alpha}$	0.10 ± 0.01 ^{b}	0.11 ± 0.01 ^{b}	0.10 ± 0.01 $^{\beta}$	0.10 ± 0.01 ^{a}	0.11 ± 0.02 ^{b}
C16:0	97.72 ± 5.20 $^{a,\alpha}$	96.49 ± 2.62 ^{a}	96.97 ± 7.22 ^{a}	95.61 ± 5.36 $^{\alpha}$	99.52 ± 4.55 ^{a}	102.77 ± 6.61 ^{a}
C16:1 n-9	0.45 ± 0.02 $^{a,\alpha}$	0.44 ± 0.02 ^{a}	0.42 ± 0.03 ^{a}	0.46 ± 0.02 $^{\alpha}$	0.51 ± 0.02 ^{b}	0.49 ± 0.03 ^{b}
C16:1 n-7	4.92 ± 0.29 $^{a,\alpha}$	5.10 ± 0.17 ^{abc}	5.25 ± 0.35 ^{abc}	4.95 ± 0.28 $^{\alpha}$	5.03 ± 0.26 ^{ab}	5.53 ± 0.42 ^{c}
C17:0	0.28 ± 0.02 $^{a,\alpha}$	0.28 ± 0.01 ^{a}	0.28 ± 0.02 ^{a}	0.28 ± 0.02 $^{\alpha}$	0.31 ± 0.03 ^{a}	0.30 ± 0.03 ^{a}
C17:1	0.50 ± 0.03 $^{a,\alpha}$	0.50 ± 0.02 ^{a}	0.50 ± 0.04 ^{a}	0.51 ± 0.03 $^{\alpha}$	0.56 ± 0.04 ^{b}	0.56 ± 0.03 ^{b}
C18:0	15.67 ± 0.84 $^{a,\alpha}$	15.54 ± 0.50 ^{a}	15.58 ± 1.06 ^{a}	15.12 ± 0.86 $^{\alpha}$	15.18 ± 0.69 ^{a}	15.40 ± 0.97 ^{a}
C18:1 n-9	660.19 ± 35.30 $^{a,\alpha}$	649.98 ± 21.45 ^{a}	643.28 ± 47.38 ^{a}	639.69 ± 35.78 $^{\alpha}$	662.01 ± 28.07 ^{a}	676.46 ± 42.20 ^{a}
C18:2 n-6	45.55 ± 2.77 $^{a,\alpha}$	46.67 ± 1.58 ^{a}	48.08 ± 3.41 ^{a}	50.51 ± 2.84 $^{\beta}$	56.97 ± 2.56 ^{b}	53.29 ± 3.43 ^{b}
C18:3 n-3	4.39 ± 0.26 $^{a,\alpha}$	4.35 ± 0.14 ^{a}	4.32 ± 0.30 ^{a}	4.47 ± 0.25 $^{\alpha}$	4.82 ± 0.21 ^{b}	4.80 ± 0.30 ^{b}
C20:0	2.46 ± 0.13 $^{a,\alpha}$	2.42 ± 0.07 ^{a}	2.37 ± 0.17 ^{a}	2.34 ± 0.13 $^{\alpha}$	2.41 ± 0.11 ^{a}	2.45 ± 0.16 ^{a}
C20:1 n-9	1.73 ± 0.09 $^{ab,\alpha}$	1.71 ± 0.06 ^{ab}	1.67 ± 0.12 ^{a}	1.71 ± 0.10 $^{\alpha}$	1.84 ± 0.09 ^{b}	1.82 ± 0.12 ^{b}
C20:2 n-6	0.35 ± 0.04 $^{c,\beta}$	0.26 ± 0.03 ^{a}	0.31 ± 0.05 ^{bc}	0.27 ± 0.03 $^{\alpha}$	0.29 ± 0.03 ^{ab}	0.27 ± 0.03 ^{ab}
C21:0	0.07 ± 0.02 $^{b,\alpha}$	0.06 ± 0.00 ^{ab}	0.05 ± 0.00 ^{a}	0.06 ± 0.01 $^{\alpha}$	0.06 ± 0.01 ^{ab}	0.06 ± 0.00 ^{ab}
C22:0	0.82 ± 0.05 $^{b,\beta}$	0.73 ± 0.03 ^{a}	0.70 ± 0.05 ^{a}	0.70 ± 0.04 $^{\alpha}$	0.74 ± 0.03 ^{a}	0.73 ± 0.05 ^{a}

C22:1 n-9	0.14 ± 0.05 b,β	0.06 ± 0.01 a	0.04 ± 0.00 a	0.05 ± 0.01 α	0.05 ± 0.01 a	0.04 ± 0.01 a
C22:2 n-6	0.27 ± 0.06 b,β	0.17 ± 0.02 a	0.14 ± 0.01 a	0.15 ± 0.02 α	0.16 ± 0.01 a	0.16 ± 0.02 a
C23:0	0.25 ± 0.11 b,β	0.14 ± 0.03 a	0.11 ± 0.03 a	0.13 ± 0.03 α	0.11 ± 0.02 a	0.13 ± 0.02 a
C24:0	0.81 ± 0.14 b,β	0.55 ± 0.04 a	0.49 ± 0.03 a	0.50 ± 0.03 α	0.52 ± 0.02 a	0.52 ± 0.04 a
Sum of FAs	836.88 ± 44.66 a,α	825.82 ± 26.68 a	820.96 ± 60.15 a	817.80 ± 45.78 α	851.49 ± 36.53 a	866.36 ± 54.46 a
SFA	118.30 ± 6.04 a,α	116.48 ± 3.29 a	116.82 ± 8.56 a	114.98 ± 6.43 α	119.13 ± 5.39 a	122.63 ± 7.85 a
MUFA	668.01 ± 35.70 a,α	657.89 ± 21.70 a	651.28 ± 47.92 a	647.47 ± 36.20 α	670.11 ± 28.39 a	685.20 ± 42.86 a
PUFA	50.57 ± 2.98 a,α	51.45 ± 1.75 a	52.86 ± 3.70 a	55.35 ± 3.16 β	62.24 ± 2.78 b	58.53 ± 3.76 b
MUFA/PUFA	13.21 ± 0.17 e,β	12.79 ± 0.05 d	12.32 ± 0.08 c	11.70 ± 0.04 α	10.77 ± 0.04 a	11.71 ± 0.03 b
oleic/linoleic	14.50 ± 0.20 e,β	13.93 ± 0.05 d	13.38 ± 0.08 c	12.66 ± 0.03 α	11.62 ± 0.04 a	12.69 ± 0.03 b

Table S2. Fatty acid composition (%). Results are expressed as mean ± standard deviation, n = 9.

FAs (%)	O1	O2	O3	O4	O5	O6
C14:0	0.02 ± 0.00	0.02 ± 0.00	0.02 ± 0.00	0.02 ± 0.00	0.02 ± 0.00	0.02 ± 0.00
C15:0	0.01 ± 0.00	0.01 ± 0.00	0.01 ± 0.00	0.02 ± 0.00	0.01 ± 0.00	0.01 ± 0.00
C15:1	0.01 ± 0.00	0.01 ± 0.00	0.01 ± 0.00	0.01 ± 0.00	0.01 ± 0.00	0.01 ± 0.00
C16:0	11.68 ± 0.04	11.69 ± 0.08	11.81 ± 0.06	11.69 ± 0.04	11.69 ± 0.07	11.86 ± 0.03
C16:1 n-9	0.05 ± 0.00	0.05 ± 0.00	0.05 ± 0.00	0.06 ± 0.00	0.06 ± 0.00	0.06 ± 0.00
C16:1 n-7	0.59 ± 0.01	0.62 ± 0.00	0.64 ± 0.01	0.61 ± 0.00	0.59 ± 0.02	0.64 ± 0.02
C17:0	0.03 ± 0.00	0.03 ± 0.00	0.03 ± 0.00	0.03 ± 0.00	0.04 ± 0.00	0.03 ± 0.00
C17:1	0.06 ± 0.00	0.06 ± 0.00	0.06 ± 0.00	0.06 ± 0.00	0.07 ± 0.00	0.06 ± 0.00
C18:0	1.87 ± 0.02	1.88 ± 0.01	1.90 ± 0.02	1.85 ± 0.00	1.78 ± 0.01	1.78 ± 0.01
C18:1 n-9	78.89 ± 0.09	78.71 ± 0.09	78.35 ± 0.09	78.22 ± 0.04	77.75 ± 0.08	78.08 ± 0.1
C18:2 n-6	5.44 ± 0.07	5.65 ± 0.02	5.86 ± 0.03	6.18 ± 0.02	6.69 ± 0.02	6.15 ± 0.01
C18:3 n-3	0.52 ± 0.01	0.53 ± 0.00	0.53 ± 0.01	0.55 ± 0.00	0.57 ± 0.00	0.55 ± 0.00
C20:0	0.29 ± 0.00	0.29 ± 0.00	0.29 ± 0.00	0.29 ± 0.00	0.28 ± 0.00	0.28 ± 0.00
C20:1 n-9	0.21 ± 0.00	0.21 ± 0.00	0.20 ± 0.00	0.21 ± 0.00	0.22 ± 0.01	0.21 ± 0.00
C20:2 n-6	0.04 ± 0.01	0.03 ± 0.00	0.04 ± 0.01	0.03 ± 0.00	0.03 ± 0.00	0.03 ± 0.00
C21:0	0.01 ± 0.00	0.01 ± 0.00	0.01 ± 0.00	0.01 ± 0.00	0.01 ± 0.00	0.01 ± 0.00
C22:0	0.10 ± 0.01	0.09 ± 0.00	0.08 ± 0.00	0.09 ± 0.00	0.09 ± 0.00	0.08 ± 0.00
C22:1 n-9	0.02 ± 0.01	0.01 ± 0.00	0.01 ± 0.00	0.01 ± 0.00	0.01 ± 0.00	0.00 ± 0.00
C22:2 n-6	0.03 ± 0.01	0.02 ± 0.00	0.02 ± 0.00	0.02 ± 0.00	0.02 ± 0.00	0.02 ± 0.00
C23:0	0.03 ± 0.01	0.02 ± 0.00	0.01 ± 0.00	0.02 ± 0.00	0.01 ± 0.00	0.01 ± 0.00
C24:0	0.10 ± 0.02	0.07 ± 0.00	0.06 ± 0.00	0.06 ± 0.00	0.06 ± 0.00	0.06 ± 0.00
